# Acute Heart Failure as a First Manifestation of Primary Adrenal Insufficiency: Highly Lethal If Not Diagnosed!

**DOI:** 10.1155/2024/5759629

**Published:** 2024-06-27

**Authors:** Maryam Heidarpour, Mohammad Mehdi Zare, Shiva Armani, Hedie Torkashvan, Sadegh Mazaheri-Tehrani, Davood Shafie

**Affiliations:** ^1^ Isfahan Endocrine and Metabolism Research Center Isfahan University of Medical Sciences, Isfahan, Iran; ^2^ Heart Failure Research Center Cardiovascular Research Institute Isfahan University of Medical Sciences, Isfahan, Iran; ^3^ Child Growth and Development Research Center Research Institute for Primordial Prevention of Non-Communicable Disease Isfahan University of Medical Sciences, Isfahan, Iran; ^4^ Student Research Committee School of Medicine Isfahan University of Medical Sciences, Isfahan, Iran

## Abstract

**Background:**

Primary adrenal insufficiency is an uncommon condition that manifests as nonspecific symptoms such as fatigue, weight loss, salt craving, and hyperpigmentation. Common cardiovascular presentations of AI are hypotension, arrhythmias, and syncope. However, acute heart failure is an uncommon presentation. *Case Presentation*. Here, a 26-year-old man was hospitalized with vasopressor-resistant cardiogenic shock, which was finally attributed to an adrenal crisis. His past medical history was notable for Hashimoto's disease, controlled with oral levothyroxine.

**Conclusion:**

AI should be considered among patients with cardiogenic shock who are unresponsive to conventional inotropes. Additionally, a history of autoimmune diseases may increase the suspicion of AI. Although the presentation of cardiogenic shock in a patient with undiagnosed AI is considered a rarity, delay in prompt treatment can lead to life-threatening conditions.

## 1. Introduction

Primary adrenal insufficiency (PAI), also known as Addison's disease, is an endocrine disorder related to insufficient production of glucocorticoid and mineralocorticoid hormones by the adrenal gland [1]. The prevalence of PAI is estimated at 82–144 cases per million, with a preponderance in females. The most prevalent etiology is autoimmune adrenalitis. PAI typically presents with symptoms of hypotension, progressive fatigue, gastrointestinal complaints, hyperkalemia, hyponatremia, and mild metabolic acidosis [1, 2]. Due to the nonspecific symptoms of cortisol deficiency, particularly in the early stage, PAI may be undiagnosed. Therefore, latent AI should be suspected in the presence of unexplained health complaints related to stress, such as fatigue, weight loss, and gastrointestinal symptoms, especially in patients with a history of autoimmune diseases, such as Hashimoto's thyroiditis [3].

Regarding cardiovascular symptoms, PAI can manifest as hypotension, arrhythmias, and syncope. However, acute heart failure (HF) is a rare but serious complication of this condition [4, 5, 6, 7, 8]. Here, we describe a case of PAI associated with cardiogenic shock unresponsive to conventional inotropes that were dramatically treated with corticosteroids.

## 2. Case Presentation

A 26-year-old man presented to our hospital in June 2023 complaining of weakness, fatigue, and dizziness starting from the morning of admission day, which progressed to loss of consciousness after a few hours. The patient had progressive dyspnea on exertion, weight loss (approximately 20 kg in the previous year), fatigue, anorexia, frequent abdominal pain, nausea, and vomiting over the past 6 months. His past medical history was notable for hypothyroidism due to Hashimoto's thyroiditis, diagnosed 6 years ago, and he was on levothyroxine. He craved lemon juice and salt. He had not been taking any over-the-counter supplements. The family history of the patient was negative for early myocardial infarction, HF, and sudden cardiac death.

Upon admission, vital signs were followed: blood pressure of 80/40 mmHg, pulse rate of 117 beats per minute, respiratory rate of 18 breaths per minute, temperature of 36.8°C, oxygen saturation of 98%, body weight of 68 kg, and height of 175 cm. On physical examination, there was abdominal pain in the epigastric region and hyperpigmentation on both flanks. The jugular vein was not distended, and there was no observed swelling in the lower limbs. The heart and lung sounds were normal on auscultation. ECG showed sinus tachycardia with no ST-T change. Due to progressive dyspnea on exertion, transthoracic echocardiography (TTE) revealed a left ventricle ejection fraction (LVEF) of 20%, mild tricuspid regurgitation, and mild pericardial effusion.

Laboratory data showed a low sodium level of 130 mmol/l (normal, 135–145 mmol/l), a high potassium level of 6.6 mmol/l (normal, 3.5–5.5 mmol/l), a high creatinine level and normal blood glucose, and white cell counts. Additionally, the troponin level was high at 17.2 ng/ml (normal, up to 0.04 ng/ml) (Table 1). A urine drug screen was negative. Venous blood gas analysis showed mild metabolic acidosis. Blood tests for infection workup were negative. Coronary angiography was performed and revealed normal epicardial coronary angiography. There was not any specific finding in the echocardiography. Additionally, abdominal and pelvic CT scans were conducted to rule out bilateral adrenal injury, hemorrhagic infarction, and metastases and it was normal.

Norepinephrine was initiated due to suspected cardiogenic shock, but the patient's hypotension persisted despite inotropes and intravenous hydration. Considering the patient's persistent hypotension, progressive fatigue, previous autoimmune disease, and laboratory data, AI was suspected. Therefore, as his case was suspicious for AI, hydrocortisone was administered at a dose of 100 mg intravenously after taking a blood sample for random plasma cortisol and adrenocorticotropic hormone (ACTH) level measurement. His clinical status and hemodynamics greatly improved. The ACTH level was 736 pg/ml (normal, 7.1–56 pg/ml), while the cortisol level was 0.23 *µ*g/dl (normal, 5–25 *µ*g/dl). With the diagnosis of PAI confirmed, the patient received 50 mg IV hydrocortisone every 6 hr. As the patient's condition improved, IV hydrocortisone was gradually tapered. When the dose of IV hydrocortisone reached below 50 mg/day, it was replaced with prednisolone (7.5 mg/day) and fludrocortisone (100 *µ*g/day). Biochemical data at discharge are given in Table 1. Predischarge TTE showed an LVEF of 55%, normal LV size, mild tricuspid regurgitation, and no pericardial effusion. He was followed in an outpatient setting regularly. Three months after discharge, the patient had no evidence of the disease. He felt well and satisfied. His blood pressure was 120/70 and he did not have orthostatic hypotention. Laboratory values were notable for serum sodium of 138 mmol/l and serum potassium of 4.2 mmol/l (Table 1). The latest TEE demonstrates the LVEF to be 55%, and other echocardiographic measurements are within the normal values. At the last visit, his weight was increased to 75 kg.

## 3. Discussion

PAI, or Addison's disease, is an endocrine disorder related to the adrenal gland's insufficient production of glucocorticoid and mineralocorticoid hormones [1]. Autoimmune adrenalitis, infections, hemorrhage, metastases, and bilateral adrenalectomy may be the etiologies of PAI. The hallmark clinical features of PAI encompass weight loss, fatigue, anorexia, salt craving, skin hyperpigmentation, abdominal pain, hyperkalemia, hyponatremia, and mild metabolic acidosis [2]. However, some patients may come with hypotension, shock, and coma, known as adrenal crisis, which can be life-threatening and needs prompt diagnosis and intervention [2, 4].

Cardiovascular manifestations of PAI are hypotension, syncope, and arrhythmia. However, acute HF as a clinical presentation of PAI is rare [4, 5, 6, 7]. The exact underlying mechanisms of HF in PAI are unknown. However, several factors contribute, such as reduced cardiac contractility, disturbance in vascular tone, fluid and electrolyte disturbance, and enhancement of inflammation. Furthermore, cortisol plays a prominent role in maintaining cardiovascular hemostasis by regulating the expression and activity of beta-adrenergic receptors, calcium channels, nitric oxide synthase, and endothelin 1. From another angle, cortisol is necessary for regulating the renin–angiotensin–aldosterone system (RAAS), subsequently influencing fluid and electrolyte balance, blood pressure control, and cardiac remodeling [4, 9, 10]. In general, glucocorticoids are crucial regulators that maintain the physiologic effect of catecholamines on the cardiovascular system. In this regard, in cardiogenic shock due to AI, administering inotropes, and vasopressors without corticosteroid results fail to recover the hemodynamic status [4, 8].

Aldosterone, as a mineralocorticoid hormone, is another contributing factor. It regulates sodium and potassium excretion, blood volume, and vascular tone. Furthermore, aldosterone may affect the myocardium directly by stimulating fibrosis, hypertrophy, and inflammation [4, 9, 10].

The diagnosis of AI is challenging due to nonspecific symptoms. Furthermore, the symptoms may be absent or masked because the clinician should have a high index of suspicion. Here, we have ruled out other possible reasons. Normal coronary CT angiography, no specific finding in echocardiography, and negative blood tests for infections ruled out the possibility of ischemic heart disease and infection, respectively. Also, normal abdominal and pelvic CT scans rule out bilateral adrenal injury, hemorrhagic infarction, and metastases. Indeed, based on the clinical manifestation, history of autoimmune disease, and our restriction on accessing for checking the autoimmune elements and considering this point that the most common cause of PAI is autoimmune adrenalitis, autoimmune adrenalitis was suspected as the etiology of the patient's condition.

The fundamental part of the treatment of AI is the concurrent replacement of glucocorticoids and mineralocorticoids, known as hormone replacement therapy. At first, when the patient was in shock, we wanted to utilize stress dose, thus IV hydrocortisone was administered. Then, as the patient's condition improved, IV hydrocortisone was gradually tapered. When the dose of IV hydrocortisone reached below 50 mg/day, it was replaced with prednisolone and fludrocortisone. Actually, for discharging the patient, we should prescribe oral agents. The standard medications for this condition are oral hydrocortisone and fludrocortisone [1, 4, 11, 12]. However, the dosage should be adjusted if the patient develops concomitant chronic HF. In this regard, fludrocortisone can lead to congestive HF by enhancing sodium retention and increasing blood pressure [4, 6, 7]. However, in our case, LVEF reached 55% after IV hydrocortisone, and at the 3-month follow-up visit, he had no signs or symptoms of HF.

## 4. Conclusion

In conclusion, AI should be considered among patients with cardiogenic shock unresponsive to conventional inotropes. Additionally, a history of autoimmune diseases may increase the suspicion of AI. Due to nonspecific symptoms on presentation, PAI can pose a challenge to diagnosis. Although the presentation of cardiogenic shock in a patient with undiagnosed AI is considered a rarity, delay in prompt treatment can lead to life-threatening conditions.

## Figures and Tables

**Table 1:** Laboratory data of the patient.

Parameter	At admission	At discharge	Three-month after discharge
HB (g/dl)	12.2	12.7	13.2
WBC (cells/*µ*l)	10,500	9,900	9,500
Platelets (x10^6^/l)	194,000	197,000	251,000
CRP	Negative	—	—
Na (mmol/l)	130	136	138
K (mmol/l)	6.6	4.0	4.2
BUN (mg/dl)	60	27	22
Cr (mg/dl)	3.1	1.5	1.1
Glucose (mg/dl)	90	95	89
Troponin (ng/ml)	17.2	4.8	0.03
TSH (mIU/l)	4.5	—	2.1
Cortisol (*µ*g/dl)	0.23	—	—
ACTH (pg/ml)	736	—	—

ACTH, adrenocorticotropic hormone; BUN, blood urea nitrogen; Cr, creatinine; CRP, C-reactive protein; HB, hemoglobin; K, potassium; Na, sodium; TSH, thyroid stimulating hormone; WBC, white blood cells.

## Data Availability

Data are included within the article.
